# Dental caries among Finnish teenagers participating in physical activity and diet intervention: association with anthropometrics and behavioural factors

**DOI:** 10.1186/s12903-021-01690-1

**Published:** 2021-07-06

**Authors:** Mirja Methuen, Sofia Kauppinen, Anna Liisa Suominen, Aino-Maija Eloranta, Juuso Väistö, Timo Lakka, Hannu Vähänikkilä, Vuokko Anttonen

**Affiliations:** 1 Institute of Dentistry, Faculty of Health Sciences, University of Eastern Finland, P.O. Box 1627, 70211 Kuopio, Finland; 2 Department of Oral and Maxillofacial Diseases, Kuopio University Hospital, Kuopio, Finland; 3 Medical Research Center, Oulu University Hospital and University of Oulu, Oulu, Finland; 4 Institute of Public Health and Clinical Nutrition, School of Medicine, University of Eastern Finland, Kuopio, Finland; 5 Institute of Biomedicine, School of Medicine, University of Eastern Finland, Kuopio, Finland; 6 Department of Clinical Physiology and Nuclear Medicine, Kuopio University Hospital, Kuopio, Finland; 7 Kuopio Research Institute of Exercise Medicine, Kuopio, Finland; 8 Infrastructure for Population Studies, Faculty of Medicine, University of Oulu, Oulu, Finland; 9 Research Unit of Oral Health Sciences, University of Oulu, Oulu, Finland

**Keywords:** Adolescent, Body mass index, Dental caries, Diet, Oral health behaviour

## Abstract

**Background:**

An association between childhood anthropometric measurements and dental caries is conflicting. The prevalence and severity of dental caries and its association with anthropometric and behavioural factors, were investigated among Finnish teenagers.

**Methods:**

The study sample comprised 202 15–17-year-old participants in the Physical Activity and Nutrition in Children (PANIC) Study. Dental caries findings were recorded using International Caries Detection and Assessment System (ICDAS) criteria, including activity estimation; numbers of decayed teeth (DT) and decayed, missing and filled teeth (DMFT) were recorded. Body weight, height and waist circumference were measured and respective body mass index (BMI) was calculated. Body fat percentage was assessed by dual-energy X-ray absorptiometry. Health-related behaviours and consumption of food and drinks were assessed using questionnaires, and intake of nutrients using a 4-day food record.

**Results:**

Mean DMFT for all the participants was 2.4 (SD = 2.9), DT 0.6 (SD = 1.3), and 36% had DMFT = 0. No difference between genders was observed. In bivariate analyses, use of sugar-sweetened beverages (SSB) three times or less per week and not having used snuff associated significantly, whereas higher carbohydrate intake (E%), toothbrushing less often than twice a day and higher caries experience at baseline almost significantly with DT > 0. In adjusted regression analyses, frequent use of SSB and higher carbohydrate intake increased the odds for DT > 0. Additionally, higher carbohydrate intake (E%) and infrequent tooth brushing significantly associated with a higher number of DT.

**Conclusion:**

Caries prevalence is still low and similar in Finnish teenage girls and boys. Behavioural factors are, but anthropometric factors are not associated with dental caries.

## Background

In 2017, oral diseases affected approximately 47% of the world’s population. About 31% had untreated caries in permanent teeth and half a billion in primary dentition [1]. World population in 2017 was 7.5 billion and in 2020 it was 7.8 billion [2]. Caries exists everywhere, although untreated caries is more prevalent in the developing world. In Finland, the mean number of decayed, missing and filled teeth (DMFT) for 12-year-olds fell from 6.9 to 1.2 between 1975 and 1994, and a similar trend was seen in the other age groups of children and adolescents [3]. Since then, a plateauing in the decline of caries has been seen [4, 5]. WHO school oral health surveys provide data on health behaviour every second year, but since the end of the 1990s there is no evidence-based data available on the prevalence of dental caries among Finnish children and adolescents.

Anthropometry refers to “the measurement of the size, weight, and proportions of the human or other primate body” [6]. Anthropometric measurements such as height and body mass index (BMI) are important indicators of children’s growth and development and they are correlated with children’s nutritional status [7]. Children’s overweight and obesity are common in today’s industrialized world, even though the increasing trend has somewhat plateaued [8]. Overweight or obesity have increased in 12–18-year-old Finnish adolescents up to two–threefold between 1997 and 2003 [9]. In 2018, nearly 25% of Finnish children and adolescents were overweight, boys more often than girls [10]. Caries and obesity are both multifactorial diseases sharing similar risk factors such as a harmful diet, socioeconomic conditions [11] and lifestyle [12]. Literature on the association between overweight and caries is contradictory; both high and low BMI showing an association with decaying teeth in children [13, 14]. The association depends on the country’s stage of development as well as on socioeconomics [13]. In Hooley’s et al. [13] systematic review, a positive association was found primarily in highly developed countries such as in Europe and the United States, whereas an inverse association or association of higher number of decayed teeth with lower BMI was found in Asia and South America. Further, Li et al. [15] in their systematic review concluded that the evidence for an association between childhood anthropometric measurements and dental caries is still conflicting and remains inconclusive.

Dietary habits have changed over the past decades. Snacking has replaced meals and beverages are consumed more frequently than ever [16, 17]. Frequent snacking and intake of sweet products are associated with overweight [18] as well as with dental caries [19]. Snacking is also associated with other harmful oral and general health habits such as smoking, snuffing and infrequent tooth brushing [20]. The difference between the genders in caries prevalence of Finnish school children appears to be disappearing [21], but girls still have slightly better oral health behaviours than boys in terms of use of sugar-sweetened beverages (SSB) and tooth brushing frequency [22, 23].

This paper aims to bring new information on caries experience and its distribution between genders. Physical Activity and Nutrition in Children (PANIC) study cohort gives an opportunity to study anthropometrics and behavioural factors in association with dental caries. The hypotheses were that boys have more dental caries than girls, and anthropometrics and high sugar intake as well as infrequent tooth brushing are associated with a higher prevalence of dental caries.

## Methods

### Study design and study population

**Fig. 1:**
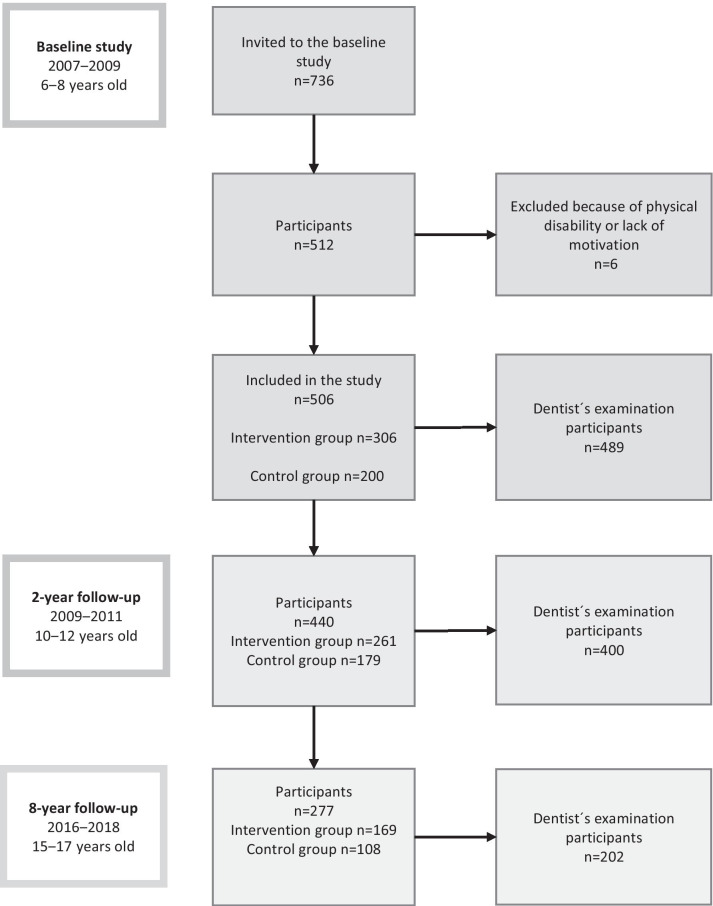
A flow-chart presenting the participants at baseline, at 2-year and 8-year follow-ups of the Physical Activity and Nutrition in Children (PANIC) study

The present analyses are based on data from the Physical Activity and Nutrition in Children (PANIC) study, which is a controlled physical activity and dietary intervention study with an ongoing follow-up in a population sample of children from the city of Kuopio, Finland*.* Altogether 736 children of 6–8 years of age who started the first grade in primary schools in Kuopio between 2007 and 2009 were invited to participate in the study. The participants were divided into intervention and control groups. The intervention included physical activity and dietary counselling sessions during the follow-up (0.5, 1.5, 3, 6, 12, 18, 24, 36, 48, 60, 72, 84, and 96 months after baseline) [24]. The children and their parents in the control group received general verbal and written advice on health improving physical activity and diet at baseline but no active intervention. The protocol of the study is described in Fig. 1. At baseline and at 2-year follow-up, dental examination was done as suggested by the WHO. Dental examination at 8-year follow-up is described in the next chapter. Public health dentists recorded teeth as having initial lesions (I), being decayed (D), missing (M) or/and having fillings (F). This protocol has been found to be reliable [25].

Data for the present analyses were obtained from the 8-year follow-up study of 202 children between 2016 and 2018. The study group consisted of 48% girls and 52% boys, the mean age (SD) being 16.5 (0.5) years.

### Assessment of dental caries and need for restorative treatment

At 8-year follow up, participants were called to a separate appointment for the dental examination. Dental examinations were carried out in a dental office at the University of Eastern Finland by an experienced dentist (MM), specialist in cariology and endodontics. MM performed all the examinations except five, which were done by a senior researcher VA with the same field of expertise. The research dentist (MM) was trained and calibrated for examinations by VA familiar with such trainings (VA) [26, 27]. Training on the study protocol was organised and criteria were reviewed using a PowerPoint presentation. Images of extracted teeth were used to demonstrate different stages of dental caries lesions according to the International Caries Detection and Assessment System (ICDAS) [28]. During the training session the senior researcher monitored MM’s work for two days (6 patients) to calibrate and verify the protocol of the clinical examination. Training and calibration were repeated every three months. To investigate the reliability of the examinations, parallel examinations were carried out (n = 13) during training sessions and inter-examiner agreement was calculated (kappa). Inter-examiner agreement was moderate with Cohen kappa value 0.574 (min 0.293, max 0.811).

Dental caries lesions were examined visual-tactilely from five tooth surfaces of all except wisdom teeth after drying them with a three-in-one syringe, using light of the unit, an oral mirror, a ball-point dental probe (WHO). Fibre-optic transillumination (FOTI) was used routinely to transilluminate the tooth surfaces (KaVo, Biberbach, Germany). MM used loops during examinations. Caries status was recorded by a dental nurse manually on a predesigned form. If there were any obstacles hindering visibility on the tooth surface (e.g. swollen gingiva, orthodontic appliances), the information was not recorded. Before the examination, dental plaque was removed with a probe, if necessary.

For estimating depth and activity (+ /−) of caries lesions, ICDAS criteria was used [28]. The score 0 represented a sound surface, enamel lesions comprised scores 1–2 and inactive score 3, lesions needing restorative treatment comprised active scores 3 and 4–6 (DT) [28]. Restorations, including materials, and missing teeth were also recorded. Simultaneously with ICDAS scores, caries activity (+/−) was recorded using the following criteria: opaque caries lesions with a whitish/yellowish appearance, a rough/soft texture of the surface after gentle probing and location in a plaque stagnation area were categorized as active lesions (+). Inactive lesions (−) were characterised by a whitish/brown/black shiny and arrested appearance, smooth/hard surface on gentle probing [29].

According to the clinical examination using ICDAS criteria, the numbers of decayed teeth (DT) and tooth surfaces (DS) and decayed, missing, and filled teeth (DMFT) and tooth surfaces (DMFS) were calculated, in order to indicate caries prevalence and severity. The prevalence of initial lesions will be reported elsewhere.

### Assessment of anthropometric factors

Body composition was assessed according to the protocol of the Panic study. Body size and composition were described as body weight (kg), body height (cm), waist circumference (cm), BMI, body mass index standard deviation score (BMI-SDS), and body fat percentage (BF%). Body weight was measured twice after overnight fasting, bladder emptied, standing upright, and in light underwear using a calibrated InBody® 720 bioelectrical impedance device (Biospace, Seoul, South Korea) to an accuracy of 0.1 kg. The mean of these two values was used in the analyses. Body height was measured three times in the Frankfurt plane without shoes by a wall-mounted stadiometer to an accuracy of 0.1 cm. The mean of the two nearest values was used in the analyses. Waist circumference was measured three times at the end of expiration at mid-distance between the bottom of the rib cage and the top of the iliac crest with a non-stretchable measuring tape to an accuracy of 0.1 cm. The mean of the two nearest values was used in the analyses. BMI was calculated as body weight in kilograms divided by body height in meters squared. Participants were categorized to normal weight, overweight and obese according to IOTF criteria [30]. BMI-SDS was computed using the national references [31]. For the analyses, BMI and BMI-SDS were further divided into equally sized tertiles based on their distributions. Mean BMI values in these tertiles ranged: lowest (range 14.9–19.6), middle (range 19.6–21.2) and highest (range 21.2–38.7). The corresponding figures for mean BMI-SDS were: lowest (range − 3.0 to − 0.4), middle (range − 0.4–0.2) and highest (range 0.2–2.8). BF% was measured bladder emptied, in supine position, in light clothing and after removing all metal objects, using a Lunar Prodigy Advance® dual-energy X-ray absorptiometry device (DXA, GE Medical Systems, Madison, Wisconsin, USA) [32].

### Assessment of health-related behaviour

The intake of energy, carbohydrates and sucrose was assessed using food records and data of the Panic study. The food records covered 4 predefined and consecutive days, including 2 weekdays and 2 weekend days or 3 weekdays and 1 weekend day. Clinical nutritionists checked the returned food records together with the adolescent, asked for any missing details e.g. about the quality and brands of the consumed products, recipes and cooking habits of the meals, and portion sizes using picture booklet of portion sizes. They defined main meals and snacks according to the recorded time and type of foods. Breakfast, lunch and dinner were classified as main meals and all eating and drinking occasions between them as snacks. Data of the number of main meals per day (< 3/3) and the number of snacks per day (< 2/2 − 3/ > 3) were recorded. Nutrient intake was calculated using the Micro Nutrica® dietary analysis software, Version 2.5. This software is based on detailed up-to-date information about the nutrient content of foods in Finland and other countries [33]. Moreover, a clinical nutritionist updated the software by adding new food items and products with their precise nutrient content based on new data in the Finnish food composition database [34] or received from the producers. The frequency of use of SSB (fizzy and energy drinks) was assessed by a questionnaire *(never/less than once a week/once a week/2 times a week/3 times a week/4 times a week/5 times a week/6 times a week/every day)*.

Tooth brushing habits were queried with following questions: How often do you brush your teeth? (*more often than twice a day/twice a day/once a day/many times per week/once a week or more seldom/never);* How do you normally brush your teeth? (*with a manual toothbrush/with an electric toothbrush/I don’t brush my teeth);* How often do you usually do interdental cleaning? *(more often than twice a day/twice a day/once a day/many times per week/once a week or more seldom/never)*. Smoking, electronic cigarette smoking and use of snuff frequency were assessed by the questions: Have you ever smoked/used electronic cigarettes/used snuff? (*never/I have done it earlier every now and then but stopped it/ I have done it earlier regularly for x (how many?) years but stopped it/I smoke every now and then but less than once a week/I smoke every now and then x (how many?) times per week/I smoke regularly every day or nearly every day).*

### Assessment of socioeconomic status (SES)

Background information was obtained from data of the Panic study and comprised household income *(EUR* ≤ *29 999/30000–59 000/* ≥ *60 000)* and parental education *(vocational/polytechnic/university)*.

### Statistics

Analyses were made using the SAS software 9.4.8 Copyright (c) 2002–2012 by SAS Institute Inc., Cary, NC, USA) and SPSS version 25 (IBM, New York, USA).

For statistical analyses, the responses from the use of SSB were categorised as follows: *once a week/2 − 3 times a week/4 − 7 times a week*. The responses to the questions concerning tooth brushing were dichotomized as follows: *at least twice a day/less often*. For smoking, electronic cigarette smoking and frequency of use of snuff the responses were: *never smoked or used/previously or currently smoking or using occasionally or frequently*. For analyses, the continuous variables BMI, BMI-SDS and carbohydrate (E%) and sucrose (E%) intakes were divided into equally sized tertiles based on their distributions.

Cross-tabulation with chi-square tests was used to analyse the differences between the categorical variables. To compare means, the independent samples T-test was used for variables with normal distributions and the Mann‐Whitney-test for variables with skewed distribution. (Tables 1, 2, 3).

**Table 1:** Characteristics of participants by gender

	All (n = 202)	Girls (n = 96)	Boys (n = 106)	*P*-value
*Group (%)*				0.702
Intervention	64	63	65	
Control	36	37	35	
Age (years), mean (SD)	16.5 (0.5)	16.4 (0.5)	16.5 (0.5)	0.378
*Socioeconomics*				
Parental education^1^ (%)				**0.028**
Vocational	13	12	14	
Polytechnic	41	51	32	
University	46	37	54	
Household income^2^ (%)				0.132
≤ 29,999€	7	5	9	
30,000–59,000€	23	29	87	
≥ 60,000€	70	67	73	
*Health-related behaviour*				
Tooth brushing frequency^3^ (%)				**0.003**
At least twice a day	73	83	64	
Less than twice a day	27	17	36	
Brushing device^3^ (%)				0.332
Electronic toothbrush	48	44	52	
Toothbrush	52	56	48	
Interdental cleaning frequency^3^ (%)				0.533
At least once a day	21	23	20	
Less than once a day	79	77	80	
Smoking^4^ (%)				0.615
Never smoker	92	93	91	
Current of former	8	7	9	
Electronic cigarette smoking^4^ (%)				** < 0.001**
Never smoker	83	95	71	
Current or former	17	5	29	
Use of snuff^4^ (%)				** < 0.001**
Never	86	95	77	
Current or former	14	5	23	
Alcohol consumption^5^ (%)				0.924
None or quitted	86	86	86	
Yes	14	14	14	
*Diet*				
Use of sugar sweetened beverages^7^ (%)				** < 0.001**
Once a week or less often	68	83	54	
2–3 times a week	22	14	31	
4–7 times a week	10	3	15	
Number of main meals per day^8^ (%)				0.878
< 3	78	78	79	
3	22	22	21	
Number of snacks per day^8^ (%)				0.253
< 2	43	37	47	
2 or 3	42	44	41	
> 3	15	19	12	
Energy intake (kcal/d), mean (SD)	1841 (511.1)	1655.9 (419.6)	2022.7 (529.2)	** < 0.001**
Carbohydrate intake (g/day), mean (SD)	216.5 (66.6)	200.9 (55.0)	231.8 (73.4)	**0.001**
Sucrose intake (g/day), mean (SD)	48.2 (25.8)	46.3 (23.3)	50.1 (28.0)	0.317
Carbohydrate (E%), mean (SD)	47.2 (7.2)	48.7 (6.2)	45.8 (7.8)	**0.007**
Sucrose (E%), mean (SD)	10.5 (5.1)	11.2 (5.1)	9.9 (5.2)	0.082
*Anthropometrics*				
Body height (cm), mean (SD)	171.2 (8.4)	165.7 (5.8)	176.1 (7.3)	** < 0.001**
Body weight (kg), mean (SD)	61.3 (11.9)	57.3 (8.3)	64.8 (13.5)	** < 0.001**
Waist circumference (cm), mean (SD)	72.7 (8.8)	69.7 (6.1)	75.4 (10.0)	** < 0.001**
Body mass index (kg/m^2^), mean (SD)	20.8 (3.3)	20.9 (2.9)	20.8 (3.7)	0.962
Body mass index SDS^9^, mean (SD)	− 0.1 (1.0)	0.01 (0.8)	− 0.2 (1.1)	0.204
Body fat (%), mean (SD)	22.6 (9.9)	28.4 (6.6)	17.3 (9.4)	** < 0.001**

*P*‐values are from independent samples T-test for variables with normal distributions or Mann‐Whitney test for variables with skewed distribution and chi‐square test for categorical variables. Bolded values indicate statistically significant associations (*P* < 0.05)

Missing data: ^1^ n = 16, ^2^ n = 20,^3^ n = 15, ^4^ n = 2, ^5^ n = 1, ^6^ n = 15, ^7^ n = 1, ^8^ n = 16

^9^ standard deviation score

**Table 2:** Caries severity and prevalence (%) by gender according to the International Caries Detection and Assessment System (ICDAS3 +)

	All (n = 202)	Girls (n = 96)	Boys (n = 106)	*P*-value^1^
Number of		Mean (SD)		
Decayed surfaces	0.63 (1.41)	0.47 (0.89)	0.77 (1.74)	0.880
Decayed, missed, and filled surfaces	4.03 (5.86)	3.39 (4.24)	4.62 (6.98)	0.389
Decayed teeth	0.59 (1.27)	0.47 (0.89)	0.71 (1.52)	0.887
Decayed, missed, and filled teeth	2.37 (2.85)	2.10 (2.34)	2.61 (3.23)	0.406

At least one		n (%)		
Decayed surface	53 (26)	26 (27)	27 (25)	0.795
Decayed, missed, or filled surface	130 (64)	58 (60)	72 (68)	0.266
Decayed tooth	53 (26)	26 (27)	27 (25)	0.795
Decayed, missed, or filled tooth	130 (64)	58 (60)	72 (68)	0.266

^1^Mann‐Whitney test, ^1^chi-squared test

**Table 3:** Distribution of participants according to number of decayed teeth (DT) (ICDAS3+) by intervention group, gender, socioeconomics, oral health-related behaviour and anthropometrics

	DT		
	0 (n = 149)	≥ 1 (n = 53)	*p*-value
*Group*			0.343
Intervention	76	24	
Control	70	30	
*Gender*			0.795
Boys	75	25	
Girls	73	27	
*Socioeconomics*			
Parental education (%)^1^			0.769
Vocational	75	25	
Polytechnic	73	27	
University	78	22	
Household income (%)^2^			0.773
≤ 29,999€	69	31	
30,000–59,000€	79	21	
≥ 60,000€	75	25	
*Health-related behavior*			
Tooth brushing frequency (%)^3^			0.066
At least twice a day	77	23	
Less often	63	37	
Smoking (%)^4^			0.846
Never	74	26	
Current or occasional	76	24	
Electronic cigarette smoking (%)^4^			0.373
Never	72	27	
Current or occasional	80	20	
Use of snuff (%)^4^			**0.038**
Never	71	29	
Current or occasional	90	10	
Smoking or use of snuff (%)			0.202
Both	89	11	
Smoking	63	38	
Snuff	90	10	
Neither	72	28	
*DIET*			
Use of sugar sweetened beverages (%)^5^			**0.033**
Once a week or less often	79	21	
2–3 times a week	69	31	
4–7 times a week	53	47	
Number of main meals per day (%)^6^			0.714
< 3	75	25	
3	73	28	
Number of snacks per day (%)^6^			0.865
< 2	75	25	
2–3	73	27	
> 3	79	21	
Carbohydrate intake (E%)^7^			
Continuous, mean (SD)	46.6 (7.4)	49.0 (6.3)	0.057
In tertiles (%)			0.147
Lowest (range 25.7–44.5)	84	16	
Middle (range 44.6–49.7)	73	27	
Highest (range 48.7–66.1)	69	31	
Sucrose intake (E%)^7^	10.3 (5.4)	11.1 (4.5)	0.185
Continuous, mean (SD)			
In tertiles(%)			0.381
Lowest (range 1.5–7.8)	82	18	
Middle (range 7.9–11.8)	73	27	
Highest (range 11.9–29.1)	70	30	
*Body composition*			
Waist (cm), mean (SD)	72.5 (8.2)	73.3 (10.4)	0.875
Weight (kg), mean (SD)	61.2 (11.4)	61.5 (13.4)	0.911
Body Mass Index			
Continuous, mean (SD)	20.8 (3.1)	21.0 (4.0)	0.963
In tertiles (%)			0.808
Lowest (range 14.9–19.6)	33	34	
Middle (range 19.6–21.2)	35	30	
Highest (range 21.2–38.7)	32	36	
BMI-SDS ^8^			
Continuous, mean (SD)	− 0.08 (0.9)	− 0.08 (1.1)	0.874
In tertiles (%)			0.980
Lowest (range − 3.0 to − 0.4)	75	25	
Middle (range − 0.4 to 0.2)	74	26	
Highest (range 0.2–2.8)	73	27	
Body fat (%), mean (SD)	21.9 (9.6)	24.5 (10.5)	0.102
*Previous caries experience*			
Number of decayed teeth at baseline, mean (SD)			
Initial or decayed lesions in deciduous or permanent teeth	1.1 (1.5)	1.7 (2.0)	0.052
Only decayed lesions in deciduous or permanent teeth	0.03 (0.2)	0.04 (0.3)	0.745
Number of decayed teeth at 2 year follow-up, mean (SD)			
Initial or decayed lesions in deciduous or permanent teeth	1.2 (1.6)	1.7 (2.0)	0.095
Only decayed lesions in deciduous or permanent teeth	0.1(0.5)	0.2 (0.5)	0.164

*P*-values are from independent samples T-test for variables with normal distributions (Carbohydrate intake (E%), Sucrose intake (E%) and BF (%)) or Mann‐Whitney test for variables with skewed distribution and chi‐squared test for categorical variables. Bolded values indicate statistically significant associations (*P* < 0.05)

Missing data: ^1^ n = 20, ^2^ n = 16, ^3^ n = 15 ^4^ n = 2, ^5^ n = 1, ^6^ n = 16, ^7^ n = 18, ^8^ = standard deviation score

Regression models were used to investigate simultaneously associations between caries and anthropometric and behavioural factors. BMI was chosen to indicate anthropometrics, and health-related behaviours (tooth brushing frequency, smoking, use of snuff and diet) based on their contribution to the occurrence of any decayed teeth (DT > 0) in bivariate analyses (Table 4). Associations were further adjusted for the PANIC intervention group, gender, socioeconomics, sucrose intake (E%) and caries severity at baseline. The occurrence of any decayed teeth (DT > 0) was used as the dependent variable in logistic regression and the number of decayed teeth in Poisson regression. The Odds Ratio (OR) and Incidence Rate Ratio (IRR) values were calculated with 95% confidence intervals.

**Table 4:** Adjusted logistic regression for occurrence of any decayed teeth (DT > 0) and Poisson regression for number of decayed teeth (DT) (ICDAS3 +)

	DT > 0 (n = 150)		DT (n = 150)	
	OR (95%CI)	*p*-value	IRR (95%CI)	*p*-value
*Group*				
Intervention	Ref		Ref	
Control	1.7 (0.7–3.9)	0.250	**1.7 (1.1–2.8)**	**0.030**
*Gender*				
Girls	1.0 (0.4–2.6)	0.980	1.0 (0.6–1.7)	0.963
Boys	Ref		Ref	
*Household income*				
≤ 29,999€	Ref		Ref	
30 000–59 000€	1.1 (0.2–7.7)	0.925	1.0 (0.3–2.9)	0.936
≥ 60,000€	1.5 (0.3–8.7)	0.636	1.0 (0.4–2.7)	0.993
*Tooth brushing frequency (%)*				
At least twice a day	Ref		Ref	
Less often	1.8 (0.6–4.9)	0.264	**2.2 (1.3–3.8)**	**0.004**
*Smoking (%)*				
Never	Ref		Ref	
Current or occasional	0.7 (0.1–5.3)	0.770	1.0 (0.3–2.7)	0.939
*Use of snuff (%)*				
Never	Ref		Ref	
Current or occasional	0.1 (0.01–1.0)	0.055	**0.3 (0.1–0.9)**	**0.028**
*Use of sugar sweetened beverages (%)*				
Once a week or less often	**0.1 (0.01–0.4)**	**0.006**	**0.1 (0.1–0.3)**	** < 0.001**
2–3 times a week	0.2 (0.02–1.2)	0.070	**0.3 (0.2–0.7)**	**0.004**
4–7 times a week	Ref		Ref	
Carbohydrate intake (E%)	1.1 (1.0–1.2)	**0.011**	1.1 (1.0–1.1)	**0.002**
Sucrose intake (E%)	0.9 (0.9–1.0)	0.260	1.0 (0.9–1.0)	0.232
Number of decayed teeth at baseline^1^	1.1 (0.8–1.4)	0.714	1.0 (0.8–1.2)	0.970
*Body Mass Index tertiles*				
Lowest (range 14.9–19.6)	0.8 (0.3–2.3)	0.633	1.1 (0.5–2.1)	0.878
Middle (range 19.6–21.2)	0.7 (0.2–2.1)	0.501	0.7 (0.4–1.5)	0.431
Highest (range 21.2–8.7)	Ref		Ref	

Bolded values indicate statistically significant associations (*P* < 0.05)

^1^Initial or decayed lesions in deciduous or permanent teeth

For the inter-examiner agreement, the Cohen-kappa value was calculated for dental caries detection between the examiner and the senior researcher on 13 patients. The kappa value was calculated as a mean of all ICDAS scores (0–6) recorded concerning occlusal surfaces in all teeth for all individuals and given here as their mean (min, max).

## Results

The study population comprised 96 girls and 106 boys, with the mean age of 16.5 (SD 0.5) years. Of the participants, 64% belonged to the intervention group. Boys were on average leaner than girls, their mean BMI being 20.8 (SD 3.3) (Table 1). According to IOTF criteria, 87.3% of the participants were of normal weight, 8.7% were overweight and 4.0% were obese, none were underweight. The total intake of carbohydrates and sucrose in grams was higher for boys than girls, whereas the opposite was true for E% of carbohydrates and sucrose. Amount of sucrose in E% exceeded the recommended 10E% in about 50% of the participants. There was no significant difference between the intervention and control groups (*p* = 0.450). Most of the participants brushed their teeth at least twice daily. Half of the participants used an electric toothbrush and one fifth performed interdental cleaning at least once a day. Electronic cigarette smoking was more common than smoking, specifically among boys (20%) (*p* < 0.001). The same was true for use of snuff. Of the boys, 23% used or had used snuff, whereas the proportion for girls was only 5%. The proportion of daily snuff users was 1% (Table 1).

Table 2 presents caries indices in the study population. On average, DMFT was 2.4 and DT comprised on average 26% of the DMFT-values. More than one third (36%) of the participants had DMFT = 0 and 74% were free of dental caries lesions. All caries indices were higher for boys than girls and the same was true for prevalence, yet the differences were not statistically significant between the genders (Table 2).

In bivariate analyses, frequent use of sugar sweetened beverages (SSB) and not having used snuff associated significantly with caries prevalence, whereas higher intake of carbohydrates (E%), infrequent toothbrushing and number of decayed teeth at baseline associated almost significantly with DT > 0. Neither gender nor any anthropometric measures were associated with present caries experience (DT) (Table 3).

In adjusted regression analyses, belonging to the intervention group rather than the control group significantly associated with a lower number of DT. Higher carbohydrate intake (E%) associated with higher odds of having any decayed teeth (DT > 0) (Table 4). The opposite was true for less frequent use of SSB compared to its daily or almost daily use. In addition, higher carbohydrate intake and tooth brushing frequency less often than twice a day significantly associated with higher number of DT or severity of dental caries. Current or previous use of snuff compared to no history of snuff use as well as less frequent use of SSB compared to daily or almost daily use were significantly associated with a lower number of DT (Table 4).

## Discussion

Caries prevalence in the present study population was low—about 25% had treatment need. Neither gender nor anthropometric measures or sociodemographic factors were associated with present caries experience (DT). The participants were on average leaner than counterparts in general population. Yet, two thirds consumed sugar sweetened beverages (SSB) weekly. Frequent use of SSB associated significantly with caries prevalence, the same was true for high carbohydrate intake (E%). High SSB consumption and carbohydrate intake and tooth brushing frequency less often than twice a day significantly associated with higher number of DT or severity of dental caries. On the other hand, receiving dietary counselling (intervention group) significantly associated with a lower number of DT compared with the controls. Use of snuff appeared to be a protective factor for caries prevalence and severity.

In 2009, DMFT for Finnish 12-year-olds was 0.7, DT 0.3 and the proportion of caries-free (DMFT = 0) was 26%. For 17-year-olds the numbers were 1.3, 0.4 and only 7.3%, respectively [4]. In 2011 DMFT for Finnish army conscripts (on average almost 20 years), was 4.1, DT 1.4 and 23.1% were caries-free. According to Finnish national statistics, approximately 60% of 12-year-olds had DMFT 0 in 2018 [21]. Offering new data on caries prevalence is a benefit of this study. Indeed, we do not have other current information on the DMFT of Finnish young people of the same age as our participants, but the trend is in line with our findings in all the studies and statistics mentioned above.

An association between childhood anthropometric measurements and dental caries has been widely studied, but the results remain inconclusive [13, 15, 35]. According to Hooley et al. [13], both high and low BMI show association with caries. The association is different depending on the country’s developmental stage and socioeconomic situation [13, 14]. Chen et al. [35] in their systematic review found that obese children from high-income countries were more likely to have a higher caries prevalence than from low-income countries. However no association between anthropometric measurements and dental caries was discovered in this study. In the PANIC study the population sample comprised predominantly healthy young teenagers. Boys and girls were slimmer compared to other Finnish adolescents in the same age group according to the National School Oral Health Survey [36]. About 13% of the participants were overweight or obese, whereas the national figure is 17%. This may explain why no association between dental caries and anthropometrics was discovered.

Frequent use of SSB was associated with presence of any decayed teeth (DT > 0) and with severity of dental caries (high DT-value). Despite the participants being part of the PANIC study and about 60% of them having received information on healthy nutrition 13 times after baseline [24], 60% of them still used SSB weekly, boys significantly more often than girls. The amount of sucrose in the diet (E%) exceeded the recommended 10E% in about half of the participants. Both the amount of sugars and the frequency of consumption are risk factors for the development of dental caries lesions [37]. Especially added sugar and sugars naturally present in honey, syrups and fruit juices and concentrates are all harmful to teeth [37]. Higher intake of sugars has also been associated with obesity [38]. The most consistent association has been between a high intake of SSB and the development of obesity seen also in children [39]. During the past decades more beverages have been consumed than ever before [16]. The findings indicated positive impact of the PANIC intervention on dental caries occurrence and further evaluation would be interesting. PANIC offers data for investigating this and the outcome could benefit for influential education.

Low SES has been associated with higher risk for dental caries in many studies [40]. However, children from high SES groups may also have an increased risk of dental caries, especially in developing countries [14]. In our study most of the participants came from families whose household income as well as parental education was high compared to the general Finnish population [41, 42]. Additionally, most had good health promoting oral and general habits. This must be born in mind while interpreting the present findings and making generalisations to the general population. However, the findings indicate behavioural patterns of teenagers concerning the use of sweet drinks, smoking and as a more recent one, snuffing.

There may be regional differences in caries occurrence. Our sample is from Kuopio City, which is the 9th largest city in Finland, with 118,000 inhabitants and situated in Eastern Finland. Both Widström and Järvinen [4] and Kämppi et al. [43] found that in Southern Finland oral health was better than in the north of the country and that more caries lesions can be detected in small rural towns than in big cities. Manifested dental caries is never a harmless condition; even low levels of dental caries in children and adolescents are of concern, because caries is a lifelong progressive and cumulative disease [44]. In our study, those who had more initial and decayed teeth at baseline also had significantly more caries experience as teenagers. However, when adjusted for behaviour and other covariates, the influence of caries history was no longer significant. The reason for this may be that prevalence of dental caries was very low even at baseline. We do not have comparable data of today’s caries situation in different parts of Finland among children and adolescents. Data collected during the 2000s is based on national patient registry data, which is not all-inclusive due to individual recall systems in municipalities.

A significant association was found between irregular tooth brushing and prevalence of dental caries lesions (DT). This still emphasizes the role of regular tooth brushing twice a day with fluoride toothpaste which has been the main reason for declining caries in the past [45]. In our study the majority, 64% of boys and 83% of girls, brushed their teeth at least twice a day which is better than on average in this age group in Finland. In the nationwide Finnish School Health Survey of 2019, only 48% of 15–16-year-old boys and 71% of girls brushed their teeth more than once a day [22]. The results show the need for oral hygiene promotion specifically targeting the boys.

We found that the use of snuff, but not smoking, was significantly associated with decreased odds for restorative treatment need due to dental caries. This is in line with a Swedish report on young adults [46] whereas in older habitual snuff users the association is not found [47]. In our study the proportion of snuff users and smokers was in line with other same-aged Finnish teenagers [48]. Of our study population, 14% used or had used snuff and 8% smoked or had smoked. It can be postulated that snuff use is a more accepted way of using nicotine products than smoking, and that snuff users may be aware of the adverse effects of snuff for oral health, which may be compensated by good oral hygiene [49].

The strength of our study is its population-based and longitudinal study design. Comprehensive data on health and health-related behavioural factors, including dietary ones, were available. Data on previous caries experience, at baseline and at the 2-year follow-up could be included in the analyses. In this second follow-up, dental caries lesions were detected carefully by a trained and calibrated senior dentist and in more detail than previously. All five surfaces of all teeth were examined using visual-tactile inspection and FOTI. However, it was not possible to take radiographs in our study. About two thirds of the participants had attended dietary counselling sessions after baseline. Due to the long follow-up period, more health-conscious teenagers may have had more predisposition to participate the 8-year follow-up.

Inter-examiner agreement was moderate. This is most likely due to the fact that agreement was calculated covering all ICDAS scores because the prevalence of caries lesions was low. Usually the cut of point sound/decayed is used in investigating inter-examiner agreement. Using that cut-off point (0/ > ICDAS 3 +) would have produced complete agreement (100%) and was therefore not used.

## Conclusion

This work provides fresh evidence-based data on the prevalence of dental caries among Finnish children and adolescents, the data at present is non-existent. Caries prevalence was low in both girls and boys, which is in line with national statistical figures. The current study suggests that behavioural, but not anthropometric factors are associated with caries experience. Indeed, healthy behaviours were important also in this low caries prevalence population. In general, the boys and girls who participated in our study had good oral and general health habits. Despite the fact that two thirds of the participants had received dietary counselling, 60% consumed sugar sweetened beverages weekly, boys more frequently than girls and sucrose consumption exceeded 10E% among half of the participants. This emphasizes the role of lifelong regular health promotion.

## Data Availability

The datasets used and/or analysed during the current study available from the corresponding author on reasonable request.
